# Fibroblast growth factor receptor-1 mediates internalization of pathogenic spotted fever rickettsiae into host endothelium

**DOI:** 10.1371/journal.pone.0183181

**Published:** 2017-08-14

**Authors:** Abha Sahni, Jignesh Patel, Hema P. Narra, Casey L. C. Schroeder, David H. Walker, Sanjeev K. Sahni

**Affiliations:** Department of Pathology, University of Texas Medical Branch, Galveston, Texas, United States of America; University of Arkansas for Medical Sciences, UNITED STATES

## Abstract

Rickettsial infections continue to cause serious morbidity and mortality in severe human cases around the world. Host cell adhesion and invasion is an essential requisite for intracellular growth, replication, and subsequent dissemination of pathogenic rickettsiae. Heparan sulfate proteoglycans [HSPGs] facilitate the interactions between fibroblast growth factor(s) and their tyrosine kinase receptors resulting in receptor dimerization/activation and have been implicated in bacterial adhesion to target host cells. In the present study, we have investigated the contributions of fibroblast growth factor receptors [FGFRs] in rickettsial entry into the host cells. Inhibition of HSPGs by heparinase and FGFRs by AZD4547 (a selective small-molecule inhibitor) results in significant reduction in rickettsial internalization into cultured human microvascular endothelial cells (ECs), which represent the primary targets of pathogenic rickettsiae during human infections. Administration of AZD4547 during *R*. *conorii* infection in a murine model of endothelial-target spotted fever rickettsiosis also diminishes pulmonary rickettsial burden in comparison to mock-treated controls. Silencing of FGFR1 expression using a small interfering RNA also leads to similar inhibition of *R*. *rickettsii* invasion into ECs. Consistent with these findings, *R*. *rickettsii* infection of ECs also results in phosphorylation of tyrosine 653/654, suggesting activation of FGFR1. Using isobaric tag for relative and absolute quantitation [iTRAQ]-based proteomics approach, we further demonstrate association of β-peptide of rickettsial outer membrane protein OmpA with FGFR1. Mechanistically, FGFR1 binds to caveolin-1 and mediates bacterial entry via caveolin-1 dependent endocytosis. Together, these results identify host cell FGFR1 and rickettsial OmpA as another novel receptor-ligand pair contributing to the internalization of pathogenic rickettsiae into host endothelial cells and the potential application of FGFR-inhibitor drugs as adjunct therapeutics against spotted fever rickettsioses.

## Introduction

Genus *Rickettsia* consists of obligate intracellular, Gram-negative bacteria, including *Rickettsia rickettsii* and *R*. *conorii*, known to be the etiologic agents of Rocky Mountain spotted fever and Mediterranean spotted fever in humans, respectively. Pathogenic spotted fever group (SFG) rickettsiae predominantly infect microvascular endothelium lining of blood vessels as their preferred primary targets in mammalian hosts, resulting in endothelial dysfunction characterized by induction of acute phase responses, infiltration of inflammatory cells, and compromised barrier function [1]. Altered vascular permeability leads to fluid leakage manifesting as edema in vital organ systems including the lungs and brain. Thus, a major theme underlying pathogenesis of these infections is rickettsial vasculitis, defined by inflammation of the microvasculature and increased vascular permeability attributed to endothelial damage/dysfunction [1, 2].

Cell surface heparan sulfate proteoglycans (HSPGs) have been implicated in cellular invasion by many bacteria [3, 4]. HSPGs are tremendously heterogeneous glycoproteins composed of a core protein and an array of sulfated repeating disaccharide units present at the cell surface and in the extracellular matrix, where they interact with multiple ligands [5]. Fibroblast growth factor (FGF)-2 requires HSPGs to interact with its tyrosine kinase receptors, namely the FGF receptors (FGFRs) [6]. Of the known FGFR subtypes [7], FGFR1 is most prevalently expressed on endothelial cells (ECs) [8], has highest affinity to FGF-2 [9], and is primarily responsible for FGF-2 induced signaling in ECs [10]. Facilitated by HS-binding motifs on both FGF-2 as well as FGFR1, downstream signaling requires formation of a ternary complex composed of FGF-2, FGFR1, and HSPGs [11]. Interactions between FGFR and FGF ligands trigger receptor dimerization resulting in juxtaposition of their intracellular tyrosine kinase domains allowing for the activation of kinase by phosphorylation of specific tyrosine residues Y463, Y583, Y585, Y653, Y654, Y730, and Y766 in the cytoplasmic domain in a precisely ordered manner. It has been demonstrated that autophosphorylation of Y653 and Y654 in the activation loop of the protein tyrosine kinase domain is critical for the FGFR1 function [12]. Resultant activation of FGFR1 leads to the recruitment and subsequent tyrosine phosphorylation of the docking protein FGFR substrate (FRS)-2α linking the receptor to intracellular signaling pathways [10].

Cell surface HSPGs have been implicated to serve as co-receptors for adherence and entry of intracellular bacteria into host cells [3, 4]. Recently, *Chlamydia trachomatis* has been shown to utilize host cell FGFR pathway to enhance bacterial infection and spread [13]. In the present study, we have investigated the involvement of HSPG-associated FGFR1 in rickettsial internalization into host ECs. Our findings suggest that SFG rickettsiae interact with the HSPG/FGFR1 complex for subsequent host cell internalization via FGFR1/caveolin-1-mediated endocytosis. Using a proteomics-based approach, we have identified β-peptide of rickettsial OmpA as an interacting partner of host FGFR1. These results demonstrate the importance of rickettsial interactions with FGFR1 in facilitating host cell invasion and substantiate the concept of exploitation of redundant entry mechanisms by pathogenic rickettsiae. Further, a small-molecule FGFR inhibitor AZD4547 exhibits a significant inhibitory effect on rickettsial invasion of microvascular endothelium and pulmonary rickettsial burden in a mouse model of infection, suggesting inhibition of FGFR1-mediated pathogen entry into host cells as a useful adjunct strategy to combat rickettsial infections.

## Materials and methods

### Cell culture and infection

Human dermal microvascular endothelial cells (ECs), obtained from the Centers for Disease Control and Prevention (Atlanta, GA), were cultured in MCDB131 medium containing 10% fetal bovine serum (Aleken Biologicals), 10 ng/ml epidermal growth factor (Thermo Fisher), 1 μg/ml hydrocortisone (Sigma), and 10 mM L-glutamine (Thermo Fisher). The host cell-free preparations *R*. *conorii* (Malish 7) and *R*. *rickettsii* (Sheila Smith) were prepared from infected Vero cells by differential centrifugation and kept frozen at -80^0^ C as small aliquots of ≤ 500 μl. The infectivity titers of these stocks were determined by quantitative PCR and plaque formation assay [14, 15]. ECs were infected with *R*. *conorii* or *R*. *rickettsii* at an approximate MOI of 1:5.

### Treatment of ECs with heparinase or FGFR inhibitor AZD4547

Heparinase (Sigma) was reconstituted in 20 mM Tris-HCl; pH 7.5, 50 mM NaCl, 4 mM CaCl_2_, and 0.01% BSA. The stock solutions of AZD4547 (Selleckchem) were prepared by solubilization in DMSO. ECs were treated with heparinase or AZD4547 and the corresponding vehicle for 1 hour at 37°C and then infected with *R*. *rickettsii*. Briefly, the cell monolayers were covered with the inoculum containing rickettsiae in a minimal volume of culture medium and the contact was enhanced by gentle side-to-side rolling of the flasks for about 15 minutes. After 6 hours, cells were washed and scraped into ice-cold PBS and processed for DNA isolation using Qiagen DNeasy Blood and Tissue Kit. Quantitative real-time PCR (q-PCR) was performed using primer pair RR190.547F and RR190.701R specific for outer membrane protein A (OmpA) of SFG rickettsiae and rickettsial copy number was calculated using a standard curve generated by the pCR2.1-TOPO plasmid containing the RR190.547/RR190.701 PCR amplicon as described earlier [16].

### Mouse model of infection and *in vivo* effect of AZD4547

Animal experiments were performed strictly in accordance with the protocol approved by the Institutional Animal Care and Use Committee at the University of Texas Medical Branch. The University has a file with the Office of Laboratory Animal Welfare, and an approved Assurance Statement (#A3314-01). C3H/HeN mice (Harlan Sprague Dawley) were intravenously infected with 2.25 x10^5^ plaque forming units (PFUs) of *R*. *conorii* per animal. AZD4547 was dissolved in a solution of DMSO/Tween-80 (1% v/v) and administered at 25 mg/kg body weight by oral gavage at the time of infection and once daily post-infection. Mice were euthanized on day 3 and the lungs were aseptically removed to be stored in an RNAlater® solution at -20^0^ C. Briefly, the animals were anesthetized using inhalational isoflurane (0.25 to 3.0%; to effect) and surgical plane of anesthesia was ensured by the absence of pedal reflex. The lungs were then removed under sterile conditions and animals were sacrificed by exsanguination. To determine rickettsial copy number, DNA isolated from the lungs using Qiagen DNeasy Blood and Tissue Kit was subjected to quantitative PCR as described above.

### FGFR1/FGFR2 knock-down

ON-TARGETplus smart small-interfering RNA (siRNA) pools for FGFR1 and FGFR2 along with a standard negative siRNA control (Thermo Fisher) were transfected into ECs using Lipofectamine RNAimax™ according to the manufacturer's recommendations. Seventy two hours later, cells were infected with *R*. *rickettsii* and subjected to the isolation of DNA for subsequent determination of rickettsial copy number.

### Immunoprecipitation and Western blotting

ECs were infected with SFG rickettsiae for 1 hour and whole cell lysates were prepared using modified radio immunoprecipitation assay (RIPA) buffer supplemented with a protease inhibitor cocktail (Cell Signaling). Immunoprecipitation (IP) was carried out using an FGFR1 antibody (Abgent) covalently cross-linked to protein G-coated magnetic beads (Thermo Fisher) using mouse IgG as a negative control. The beads were then washed thoroughly with RIPA buffer and the samples thus prepared were analyzed by SDS gel electrophoresis and Western Blotting using an OmpA antibody (kindly provided by Dr. Donald H. Bouyer, UTMB) at 1:1,000 dilution. In other experiments, caveolin-1 or caveolin-2 (Cell Signaling), phospho-FGFR1 (Y653/654, Cell Signaling), and α-Tubulin (Accurate Chemical and Scientific) antibodies were used in conjunction with appropriate HRP-conjugated secondary antibodies.

### Gene expression analysis by quantitative real-time PCR

Total RNA isolated from infected ECs using TRI^®^ Reagent (Molecular Research Center) was subjected to preparation of cDNA employing a high capacity cDNA synthesis Kit (Thermo Fisher). PCR reactions were performed in a StepOnePlus™ thermal cycler (Applied Biosystems). Target gene expression was normalized to GAPDH and relative expression was calculated by ^ΔΔ^*C*_T_ method as described previously [17].

### Mass spectrometry

ECs were infected with SFG rickettsiae for varying duration of time (up to 1 hour). Total protein lysates were prepared for IP of FGFR1 as described above. The samples were processed further using a 4-plex iTRAQ (isobaric tag for relative and absolute quantitation) labeling kit according to the manufacturer’s protocol (AB SCIEX LLC). In this approach, all N-terminus and side chain amines of the peptides from digested protein are covalently labeled with the tags of varying molecular mass, fractionated by chromatography, and analyzed by mass spectrometry. The labeled peptides were then subjected to the NCBI database search to identify the rickettsial protein(s).

### Statistical analysis

Each experiment was performed at least three times and the results are presented as the mean ± standard error (SE) unless otherwise stated. For comparison among experimental conditions, one/two way ANOVA with Dunnett's post-test was performed using GraphPad Prism 4.00. The p value for statistical significance was set at ≤ 0.05.

## Results

**Fig 1:**
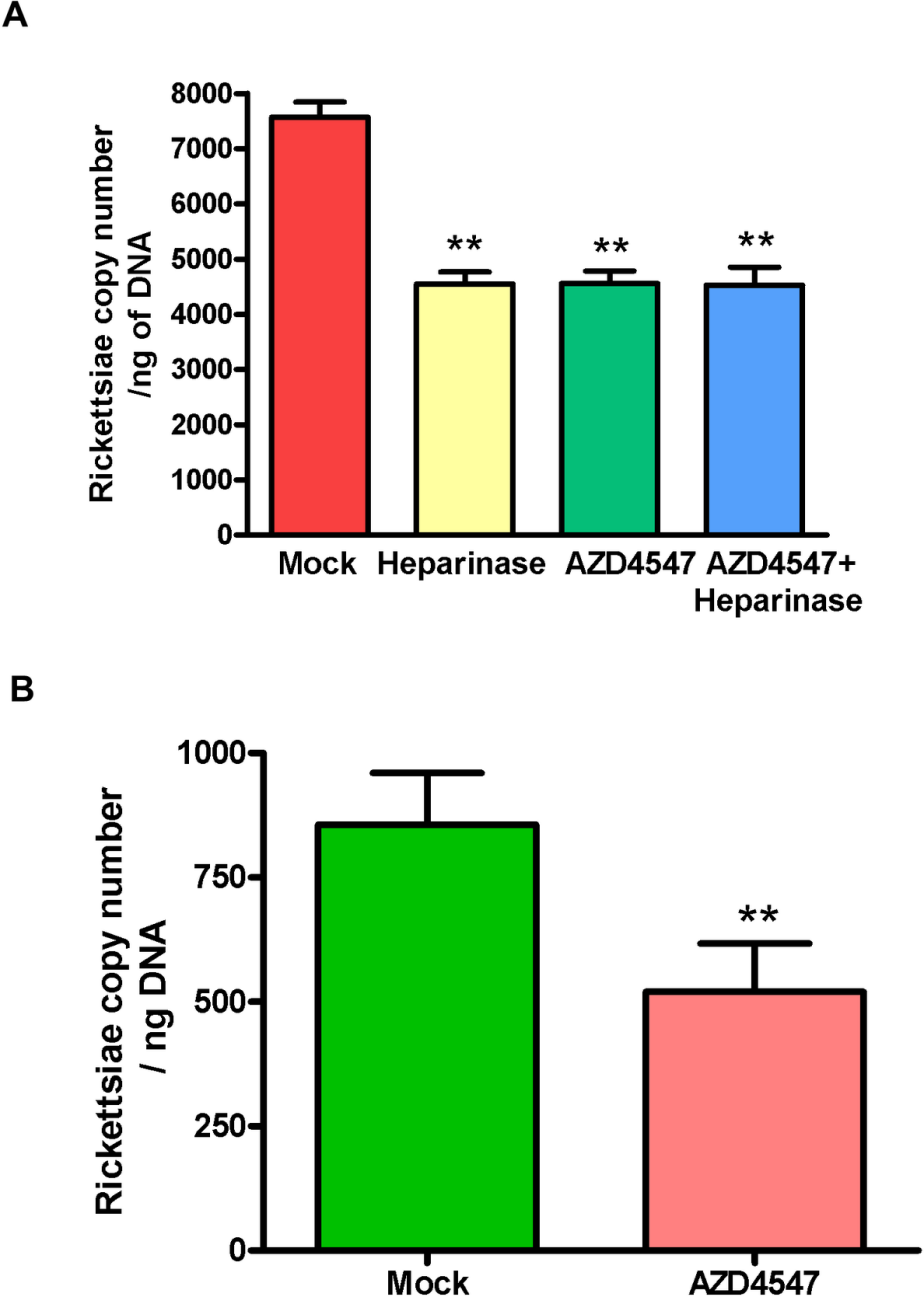
Effect of FGFR/HSPG inhibition on rickettsial internalization *in vitro and in vivo*. (A): ECs were incubated with heparinase (1U/ml), FGFR inhibitor AZD4547 (100 nM), or the vehicle alone (mock-treated) for 1 hour prior to infection with *R*. *rickettsii*. At 6 hours post-infection, total DNA was extracted and rickettsial copy number was determined using a standard curve. The asterisks represent a significant change (p ≤ 0.001) compared to mock treatment and the data represent mean ± Standard Error (SE) from a minimum of three independent experiments. (B): AZD4547 was dissolved in a vehicle containing DMSO and 1% (v/v) Tween-80 and orally administered (25 mg/kg/day) to 6–8 weeks old C3H/HeN mice infected with *R*. *conorii* (2.25×10^5^ pfu). The control group of animals (infected but mock-treated) received injection of *R*. *conorii* and vehicle (DMSO + Tween-80). Mice were euthanized on day 3 post-infection and rickettsial copy number was determined using DNA from the lungs. The data are presented as the mean ± SE of three independent observations.

To investigate the potential contributions of FGFRs in facilitating rickettsial entry into target host cells, we employed a small-molecule inhibitor AZD4547, a potent and selective FGFR tyrosine kinase inhibitor currently in phase II and III clinical trials (ClinicalTrials.gov Identifier: NCT02154490), and heparinase to cleave the sulfated side chains of heparin sulfate proteoglycans required for FGFR1 activation and dimerization [18]. HMECs were incubated with heparinase (1U/ml) or AZD4547 (100nM) along with the corresponding vehicle controls for 1 h prior to infection with *R*. *rickettsii* to determine the effects on the number of intracellular rickettsiae. Pretreatment of HMECs with AZD4547 prior to infection with *R*. *rickettsii* resulted in a reduction of 41 ± 6% in rickettsial copy number while there was 43 ± 4% inhibition with heparinase treatment, indicating the involvement of FGFRs. Also, treatment with both heparinase and AZD4547 to test the combinatorial effect also resulted in a decline of 42 ± 5% in rickettsial copy number, suggesting the involvement of FGFR and HSPGs in facilitating rickettsial internalization into ECs (Fig 1A). To confirm these findings in an independent set of experiments, HMECs were incubated with DNaseI (1U/ml) or gentamicin (100 μg/ml) for 30 minutes to remove any extracellular bacteria prior to the collection of infected cells for isolation of DNA. We did not notice any significant differences in the copy number of internalized rickettsiae with or without DNaseI/Gentamicin treatment (S1 Fig). To ensure that the observed reduction in rickettsial copy number was not due to detrimental effects of heparinase and AZD4547 treatment on the host cell viability, we also performed a quantitative lactate dehydrogenase (LDH) release assay (Thermo Fisher Scientific) on the culture supernatants as a marker of potential cytotoxicity. Our results did not suggest loss of viability or evidence of cytotoxicity after inhibitor treatments as compared to vehicle-treated controls. For all experimental conditions, the extent of cell viability was determined to be ≥ 95%, representing a loss of ≤ 5% attributed to normal turnover of cells in culture (S2 Fig).

We next investigated the role of FGFRs in rickettsial invasion in a mouse model of infection, taking into consideration that FGFR1 is the major isoform expressed by ECs *in vivo* [19]. Since pulmonary edema is a prominent pathologic feature of human rickettsioses, we measured rickettsial load in the lungs of infected mice in the presence and absence of AZD4547 treatment. Mice infected intravenously with *R*. *conorii* either received the vehicle alone or AZD4547 (25 mg/kg once daily by oral gavage on the day of infection and thereafter). The dose of AZD4547 was selected based on its potent antitumor activity against FGFR-deregulated tumors in preclinical models [20]. On day 3, lungs were processed for the isolation of DNA and determination of rickettsial copy numbers by OmpA-based q-PCR. In congruence with the *in vitro* findings using cultured ECs, there was significant reduction (40 ± 7%, p< 0.01) in the number of rickettsiae in the lungs of AZD4547-treated mice in comparison to the corresponding cohort of infected but vehicle-treated animals (Fig 1B).

**Fig 2:**
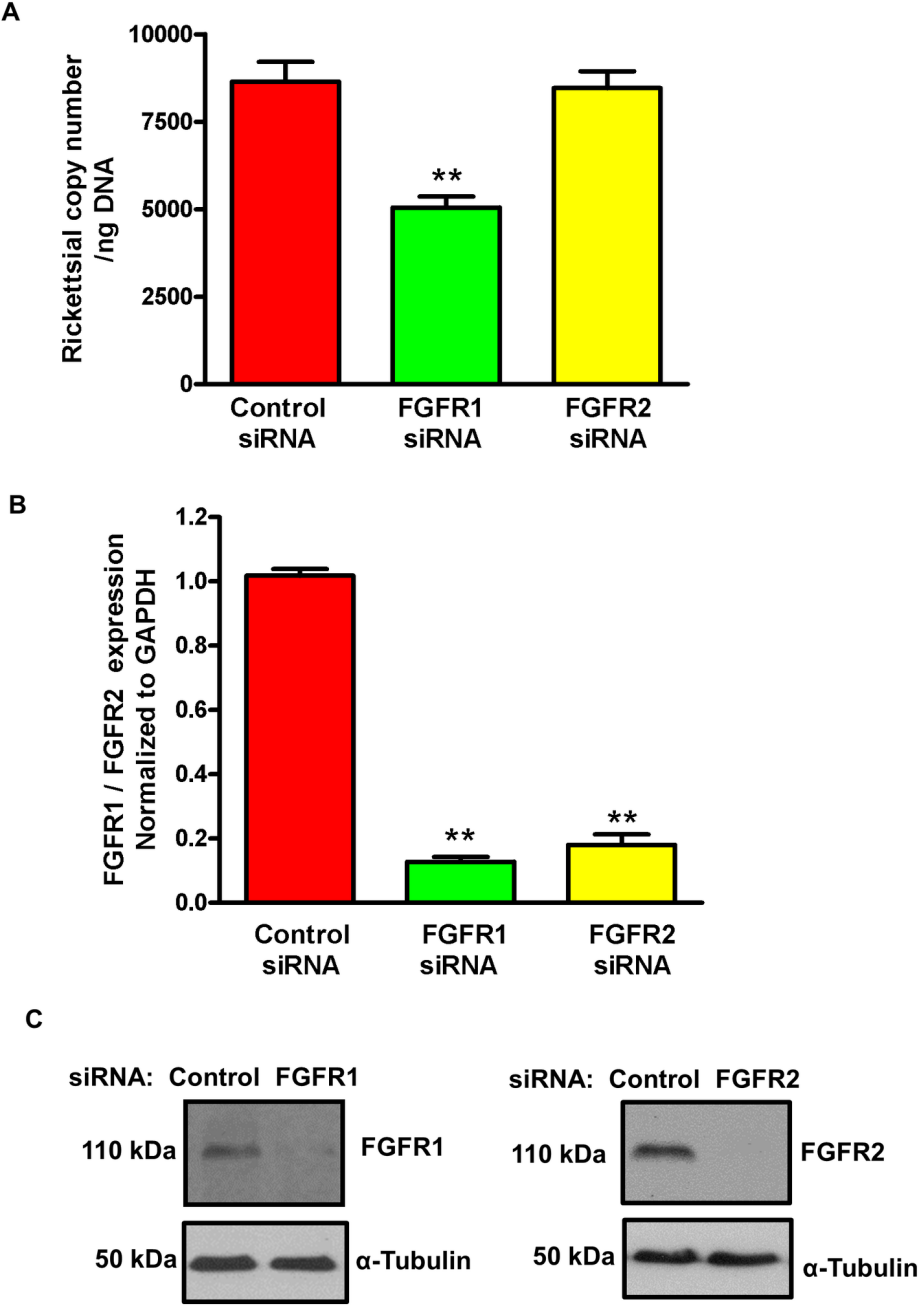
Effect of FGFR1/FGFR2 knockdown on rickettsial entry. (A): ECs were transfected with the control, FGFR1, or FGFR2 siRNAs (100nM) using Lipofectamine-RNAiMAX. After 72 hours, cells were infected with *R*. *rickettsii* and DNA was isolated to quantify rickettsial copy number by q-PCR using the OmpA primer pair. The asterisks represent a significant change (p ≤ 0.001) compared to control siRNA. The data are presented as the mean ± SE of three independent experiments. (B): Quantitative-PCR based expression of FGFR1 and FGFR2 mRNA to ensure the efficacy and specificity of siRNAs. (C): Western blot analysis for FGFR1 and FGFR2 ascertaining the efficiency of knockdown at the level of protein.

Amongst the major known isoforms of FGFRs designated as FGFR1-4, only FGFR1 and FGFR2 are predominantly expressed on ECs [21]. Therefore, we next probed whether siRNA-directed silencing of FGFR1 or FGFR2 interferes with rickettsial entry into host ECs. Again, FGFR1 knock-down resulted in the inhibition of *R*. *rickettsii* invasion by 40 ± 4%, whereas rickettsial entry was not adversely affected consequent to siRNA interference with FGFR2 expression, implicating the involvement of FGFR1 (Fig 2A). The specificity and efficacy of siRNAs used in our experiments was ensured by measuring both mRNA expression by q-PCR (Fig 2B) and the steady state levels of FGFR1 and FGFR2 protein by Western blot analysis (Fig 2C). Cumulatively, these results reveal convincing evidence for rickettsial interaction(s) with FGFR1 in facilitating the host cell invasion both *in vitro* and *in vivo*.

**Fig 3:**
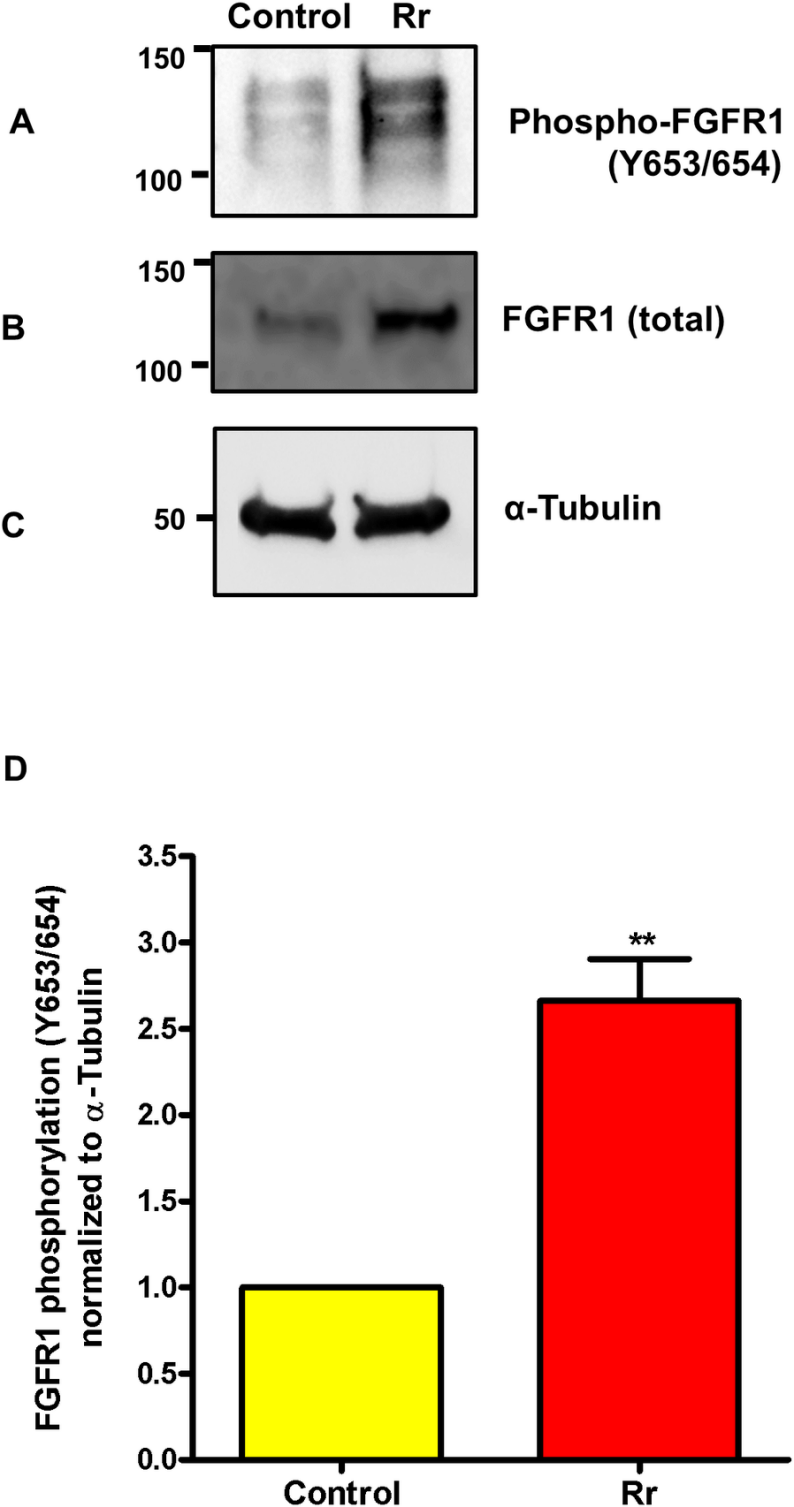
*Rickettsia* infection activates FGFR1. ECs were infected with *R*. *rickettsii* for 30 minutes and the levels of FGFR1 phosphorylation (tyrosine 653/654) (A), total FGFR1 (B), and α—tubulin as the loading control (C) were measured by immunoblotting and probing of the blots using specific antibodies. Quantitation of FGFR1 phosphorylation from three separate experiments is also presented as the mean ± standard error (D).

To further investigate whether host cell infection activates FGFR1 signaling, ECs were infected with *R*. *rickettsii* and FGFR1 phosphorylation on Y653/654 was assessed as an indicator of FGFR1 transactivation. Y653 and Y654 are located in the Src homology kinase domain and phosphorylation of these residues triggers a cascade of reactions leading to the recruitment and assembly of downstream signaling complexes, suggesting critical importance for these tyrosines in FGFR1 function. In comparison to uninfected ECs, the steady-state levels of phospho-FGFR1 were significantly higher as early as 30 minutes post-infection, indicating FGFR1 activation in response to infection (Fig 3A). A similar pattern of increase in the FGFR1 protein was also evident (Fig 3B). Quantitation of FGFR1 phosphorylation using α—Tubulin as the loading control (Fig 3C) and the levels in uninfected ECs as the baseline control suggest that *R*. *ricekttsii* infection induces about 2.7-fold increase of phosphorylation at Y653/654, yielding evidence for FGFR1 activation (Fig 3D).

**Fig 4:**
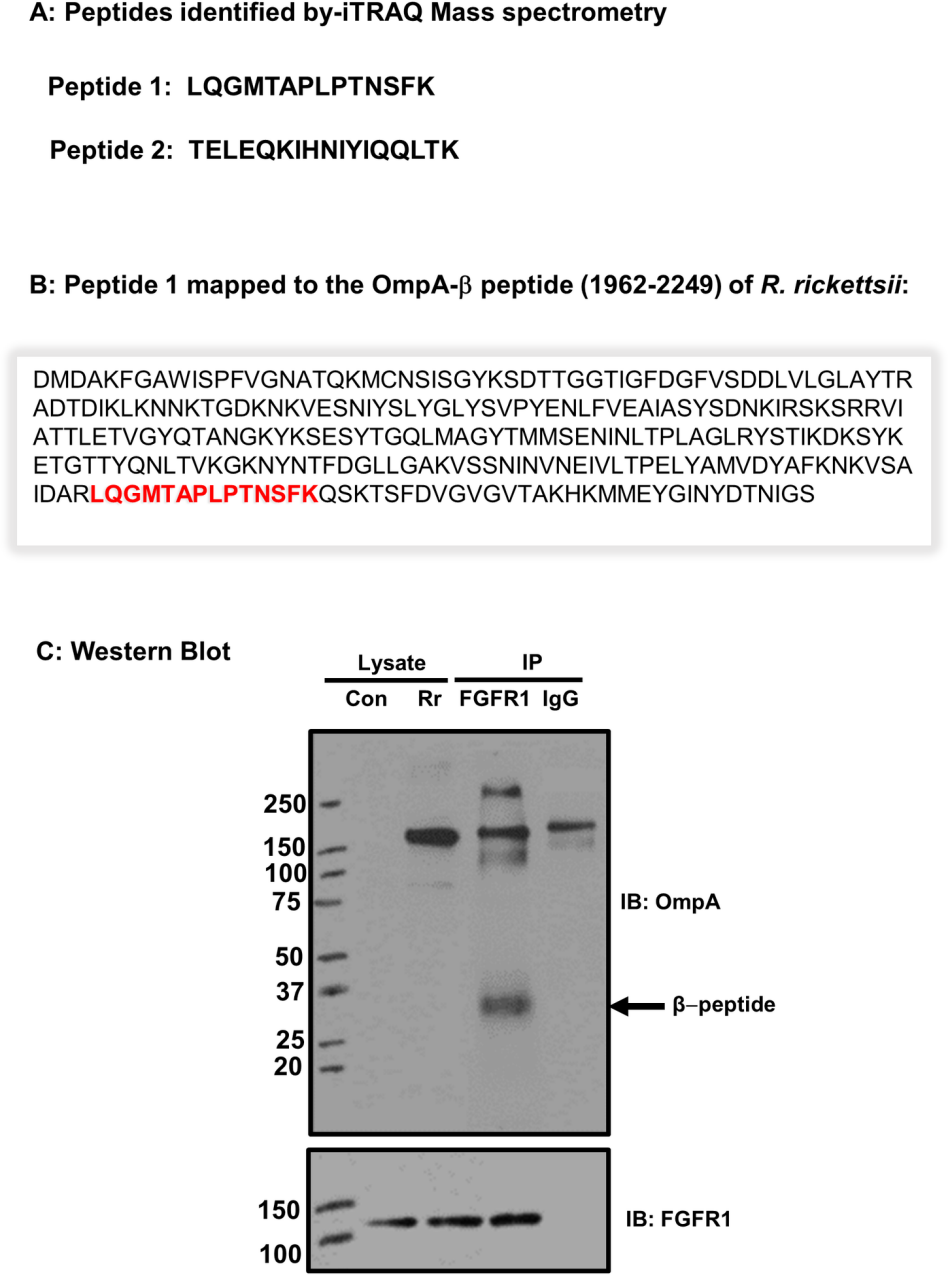
FGFR1 interacts with β-peptide of OmpA. Confluent ECs were infected with *R*. *rickettsii* and FGFR1 was immunoprecipitated from the total protein lysates. The samples were subjected to mass spectroscopic analysis using isobaric tag for relative and absolute quantitation [iTRAQ] method [described in materials and methods]. (A): The peptides interacting with FGFR1 were identified as peptide 1 and 2. (B): The location of the peptide 1 within the beta peptide sequence of OmpA is shown in red. (C): FGFR1 was immunoprecipitated (IP) from *R*. *rickettsii-*infected ECs and samples were subjected to SDS-PAGE and Western blotting using rickettsial OmpA antibody. Mouse IgG was used as the control. The blot was also probed with an FGFR1 antibody to demonstrate that the immunoprecipiatation was successful. A representative blot from three independent experiments is shown.

Next, ECs were infected with *R*. *rickettsii* for various times up to 1 hour, followed by IP using an FGFR1 antibody and mass spectrometry analysis using 4-plex-iTRAQ labeling to identify rickettsial protein(s) interacting with host FGFR1. Due to the isobaric mass design of the iTRAQ reagents, differentially labeled proteins do not differ in mass; accordingly, their corresponding proteolytic peptides appear as single peaks in MS scans. This proteomics-based approach targeting the primary amino groups for identification and relative quantitation of proteins enabled us to identify two different peptides, namely peptide 1 and peptide 2 (Fig 4A). A BLASTp search of rickettsial protein databases using these peptide sequences revealed two rickettsial proteins, OmpA and Sca2, with the likelihood of interactions with mammalian FGFR1. Interestingly, peptide 1 mapped to the β-peptide region of OmpA (Fig 4B). Further, the sequence of this peptide in OmpA of *R*. *rickettsii* and *R*. *conorii* was found to be highly conserved as suggested by 100% identity. The location of this peptide was mapped to amino acid positions 1962 to 1975 in the β-peptide region of OmpA in *R*. *conorii* and positions 2190 to 2203 for *R*. *rickettsii*. Peptide 2, on the other hand, also mapped with 100% sequence similarity to Sca2 of *R*. *conorii* (amino acid positions 1248 to 1264) and *R*. *rickettsii* (1233 to 1249). To further investigate these interactions, lysate from ECs infected with *R*. *rickettsii* was immunoprecipitated using an FGFR1-specific antibody and subjected to Western blotting using an OmpA antibody or Sca2 antiserum. Our results suggest that β–peptide of OmpA interacts with FGFR1 as evidenced by a prominent band at approximately 32 kDa (Fig 4C). Application of a similar approach did not yield evidence for interactions of *R*. *rickettsii* Sca2 with FGFR1 (S3 Fig).

**Fig 5:**
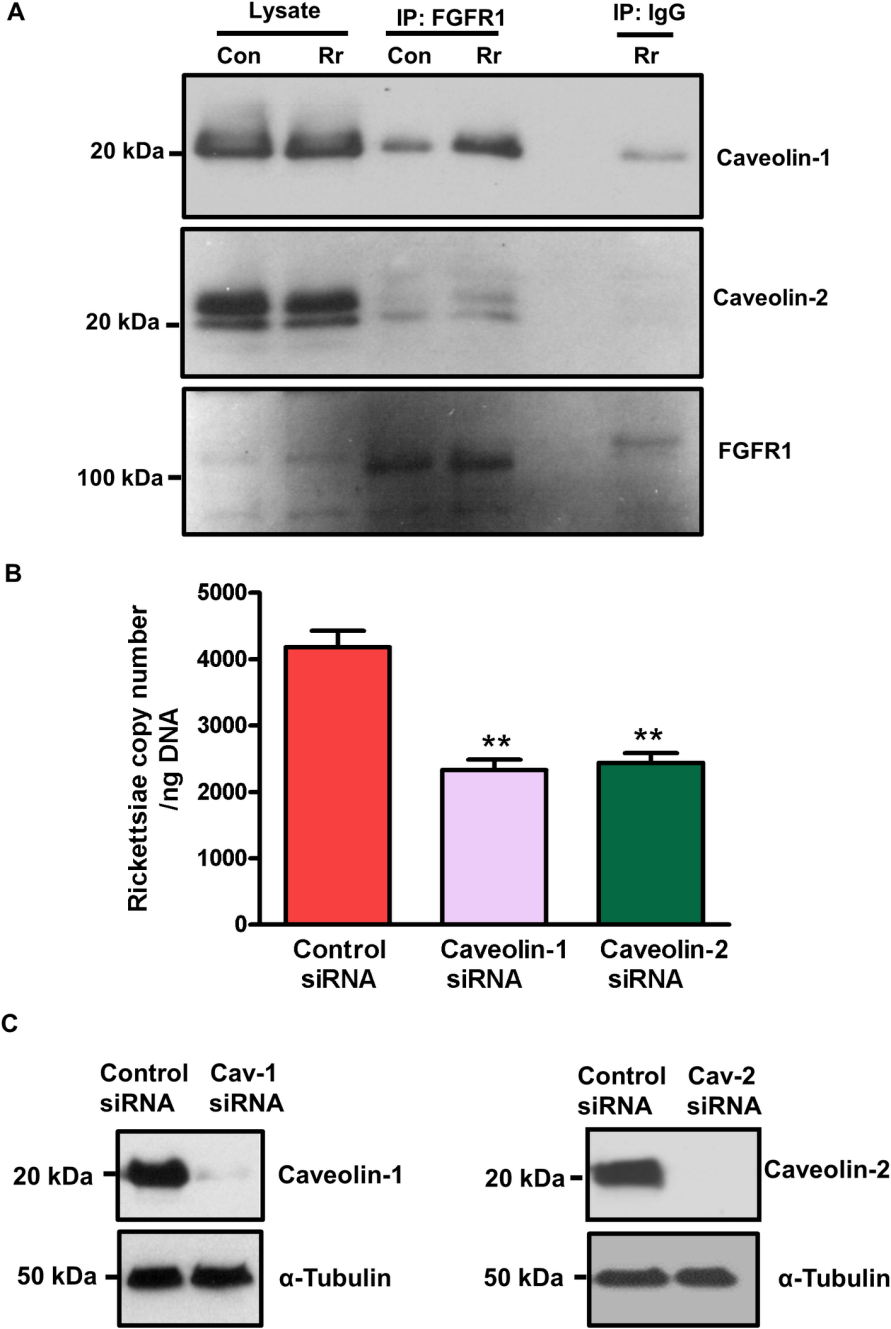
FGFR1 interactions with caveolin-1 and caveolin-2. (A): Confluent ECs were infected with *R*. *rickettsii*. At 1 hour post-infection, the cell lysates were prepared, FGFR1 was then immuno-precipitated using an FGFR1-specific antibody. Mouse IgG was used as a negative control. Samples were subjected to SDS-PAGE and Western blotting using antibodies against caveolin-1, caveolin-2 and FGFR1. (B): ECs were transfected with either control, caveolin-1 or caveolin-2 siRNA for 72 hours and then infected with *R*. *rickettsii* for 1 hour. Rickettsial copy number was measured by q-PCR using the OmpA primer pair. The asterisks represent a significant change (p≤ 0.001). The data are presented as the mean ± SE of three independent experiments. (C): caveolin-1 (cav-1) and caveolin-2 (cav-2) expression were measured by Western blotting to demonstrate the functionality of siRNAs used in our experiments.

Receptor-mediated internalization of pathogens into mammalian cells occurs via endocytosis [22] FGF-2 induces internalization of cell surface-associated FGFR1 through a caveolin-dependent endocytic pathway followed by its nuclear translocation [23, 24]. Therefore, we next determined the possibility of FGFR1 interactions with caveolin-1 and -2. Protein lysates from ECs infected with *R*. *rickettsii* were immunoprecipitated using an FGFR1-specific antibody followed by Western blot analysis for caveolin-1 and -2. The findings demonstrate that *Rickettsia* infection promotes FGFR1 binding to caveolin-1, while there apparently is no association between FGFR1 and caveolin-2 (Fig 5A). To further investigate whether or not FGFR1-mediated rickettsial entry into host endothelium is caveolin-dependent, ECs transfected with control, caveolin-1, or caveolin-2 siRNAs were infected with *R*. *rickettsii* and the copy number of internalized rickettsiae was determined. Interestingly, interference with both caveolin-1 and caveolin-2 resulted in significant reduction in the number of intracellular rickettsiae (42 ± 4%, p< 0.01), in direct comparison to cells transfected with the control siRNA (Fig 5B). The efficacy of siRNAs was ascertained by Western blotting as shown in Fig 5C.

## Discussion

Employing a coalescence of biochemical, molecular, and pharmacological approaches, we have identified that pathogenic *Rickettsia* species belonging to the spotted fever group avail the host FGFR1/HSPGs complex to gain entry into target ECs. The evidence suggests that rickettsiae interact with FGFR1 and subsequent activation of FGFR1-mediated signaling may facilitate rickettsial internalization into host cells. Such initial interchanges between invading rickettsiae and host FGFR1 signaling machinery contribute to bacterial uptake into host cells via caveolin-1 dependent endocytosis. Results also demonstrate the β-peptide of rickettsial OmpA to be an interacting partner for FGFR1. Together, these findings represent the first report on the contributions of OmpA-FGFR1 as yet another ligand-receptor system and associated downstream signaling mechanisms in facilitating rickettsial internalization into host endothelium.

Receptor tyrosine kinases are transmembrane-type receptors, which transduce extracellular signals to different intracellular signaling cascades [25]. FGFR1, FGFR2, FGFR3, and FGFR4 constitute the FGFR family of receptor tyrosine kinases with three immunoglobulin G-like domains in the extracellular region [25, 26]. A fifth receptor, FGFR5 has no tyrosine kinase domain and may negatively regulate signaling [27]. Receptor tyrosine kinase requires the presence of HSPGs [28] and formation of a ternary FGFR signaling complex, facilitated through HS-binding motifs on both the FGF ligand and the receptor tyrosine kinase [29]. Our data consistently suggest about 40% reduction in the copy number of intracellular rickettsiae irrespective of the inhibition of each individual component or together, suggesting the participation of this signaling complex involving FGFRs and HSPGs in rickettsial internalization. Importantly, ECs display much lower steady-state levels of FGFRs 2–4, but abundant expression of FGFR1 [30], and knockdown of FGFR3 and 4 does not affect endothelial morphology [31, 32], suggesting that FGFR1 is primarily expressed and involved in signaling in ECs. Thus, our data implicating the requirement of FGFR1 in rickettsial internalization is compatible with the abundance and importance of FGFR1 functions in vascular endothelium.

Multiple surface cell antigens (Scas), also known as autotransporters, are involved in rickettsial adhesion to host cell surface components. Among *Rickettsia* species, five major identified antigens are Sca0 (OmpA), Sca1, Sca2, Sca4, and Sca5 (OmpB) [33]. Of these, OmpA is conserved throughout the SFG, whereas OmpB is conserved in both the SFG and typhus group [34]. To date, two major rickettsial ligand–host receptor pairs have been implicated in the invasion of host cells, including OmpB—host cell receptor Ku70 (the subunit of a nuclear DNA-dependent protein kinase) [35] and OmpA—cell surface α2β1 integrin [36]. It is important to note that many of these interactions have been illustrated employing a heterologous system wherein rickettsial Omp being investigated is expressed at the outer membrane of *Escherichia coli*. A direct knockout of OmpA in *R*. *rickettsii* via insertion of a premature stop codon into the open reading frame using a group II intron, however, does not display an altered phenotype in cell culture as evidenced by no noticeable differences in the invasion and growth kinetics in Vero cells and maintenance of virulence in a guinea pig model of infection [37]. Among other Sca proteins, Sca1 has been demonstrated to be involved in rickettsial attachment to mammalian cells, but has no role in invasion [38], and Sca4 has been shown to co-localize with and activate host vinculin at focal adhesion sites [39]. Published evidence further suggests that Sca2 also mediates adherence to and invasion of host cells [40]. Again, application of random transposon mutagenesis to recover a small plaque mutant of *R*. *rickettsii* with disrupted Sca2 reveals an important role for this protein in actin-based motility and virulence mechanisms, but no significant changes in the rate of intracellular multiplication *in vitro* or the ability to replicate and cause seroconversion in infected guinea pigs despite reduced virulence *in vivo* [41]. On the host side, exchange protein directly activated by cAMP (Epac1) has been documented to facilitate host-pathogen interactions and rickettsial adhesion and invasion [42]. Collectively, ample evidence supports the perception that due to fastidious growth requirements and lifestyle as strict intracellular parasites, *Rickettsia* species exploit multiple redundant strategies to ensure both efficient adhesion and cellular entry to quickly achieve access to the nutrient-rich host cytoplasm for their growth and replication and interactions with FGFR1 likely represent one of these mechanisms.

We further demonstrate that FGFR1 interacts with OmpA, expressed only by the SFG rickettsiae. Interestingly, OmpA exhibits homology to a family of modular proteins termed autotransporters in Gram-negative bacteria, a majority of which are critically important for virulence [43]. Of note, there are interspecies variations in the molecular mass of OmpA among spotted fever rickettsiae. For example, *R*. *rickettsii* expresses a 247 kDa OmpA, whereas *R*. *conorii* encodes for a 224 kDa OmpA. Similar to other known autotransporters, OmpA undergoes post-translational processing and is cleaved to release a 190 kDa (or 155kDa) segment from the translocation pore (32 kDa). OmpA and OmpB share significant similarity in their C-terminal autotransporter domains, which is expressed at the bacterial surface. Recently, outer membrane proteome analysis of the virulent Sheila Smith strain of *R*. *rickettsii* by mass spectrometry has identified four OmpA peptides indicating that after post translational processing, β-fragment of OmpA exists in rickettsial outer membrane along with OmpB [44]. In our study, proteomics approach based on iTRAQ mass spectrometry identified two different peptides mapping to the β-peptide region of OmpA and Sca2 of *R*. *rickettsii* and *R*. *conorii*, suggesting the possibility of their involvement as FGFR1 interacting partners. Subsequent application of a standard molecular approach of IP/IB (immunoprecipitation coupled to immunoblotting) allowed us to further ascertain that 32 kDa autotransporter domain of OmpA interacts with FGFR1 to facilitate bacterial invasion into host cells.

FGFR endocytosis, trafficking, and signaling are critically important for the regulation of a variety of cellular functions including proliferation, differentiation, and development [24]. Published reports have described crucial roles for clathrin/caveolin-dependent endocytic machinery in the entry of *Listeria monocytogenes*, *Ehrlichia chaffeensis* and *Anaplasma phagocytophilum* into non-phagocytic cells [45–47] and for rapid and sustained ubiquitination of Ku70 via recruitment of c-Cbl in the internalization of *R*. *conorii* [35]. Caveolin-1 is one of the major structural proteins essential for the formation of caveolae in ECs [48, 49]. FGFR1 is internalized via a caveolin-1 dependent pathway [24] and caveolin-1 interacts with caveolin-2 in ECs [50, 51]. Our results imply that FGFR1 likely interacts with caveolin-1, because *Rickettsia* infection enhances the association of these proteins. An intriguing aspect of our findings, however, is significant reduction of bacterial uptake after independent silencing of caveolin-1 as well as caveolin-2. The plausible explanations for such an effect in our system are that caveolin-2 binds to caveolin-1 [51] and that rickettsiae may exploit Ku70 as another known receptor to gain entry via caveolin-2 dependent endocytosis [35].

The FGFR signaling pathway has been implicated in multiple pathologies and monoclonal antibodies as well as orally bioavailable inhibitors targeting FGFRs are being developed as potential therapeutics for different carcinomas. AZD4547 is a potent and selective small-molecule inhibitor of FGFR1 with an IC_50_ value of 0.2 nM and currently in phase II and III clinical trials for the treatment of cancer (ClinicalTrials.gov Identifier: NCT02154490). The 1.65 A° resolution crystal structure of AZD4547 bound to the kinase domain of FGFR1 has been determined and reveals AZD4547 to be a type 1 kinase inhibitor targeting the active kinase conformation of FGFR1 [52]. In the present study, a significant decline of ~40% in the pulmonary rickettsial load in AZD4547-treated, *R*. *conorii*-infected mice not only authenticates the effects seen in host cells, but also yields the first evidence for interference with FGFR1 as a potential anti-microbial strategy. As another group of obligate intracellular pathogens, *Chlamydia trachomatis* and *C*. *muridarum* have recently been shown to utilize multiple mechanisms to co-opt the host cell FGFR pathway to enhance bacterial infection and spread [13]. Also, adeno-associated virus 2, which is routinely employed as a viral vector in human gene therapy, requires human FGFR1 as a co-receptor for successful entry into the host cell [53].

In conclusion, this study reports on the identification of host cell FGFR1 and rickettsial OmpA β-peptide as another receptor-ligand pair contributing to the internalization of *R*. *rickettsii* and *R*. *conorii* into host ECs *in vitro* and the lungs as one of the major target organs *in vivo*. Our findings, thus, reveal a promising new and selective anti-rickettsial target and the amenability of repurposing of FGFR inhibitors under development as adjunct therapeutics against infections due to spotted fever rickettsiae.

## Supporting information

S1 Fig Effect of DNaseI and Gentamicin on rickettsial internalization. (PDF)

S2 Fig Effect of heparinase and AZD4547 on viability of endothelial cells. (PDF)

S3 Fig Lack of Sca2 association with FGFR1. (PDF)

S1 File Whole blots with markers. (PDF)
