# Supplementary material for: Fibroblast growth factor receptor-1 mediates internalization of pathogenic spotted fever rickettsiae into host endothelium
Source: PLoS One. 2017 Aug 14;12(8):e0183181. doi: 10.1371/journal.pone.0183181 (PMC5555671; doi:10.1371/journal.pone.0183181)
Supplement: S1 Fig — (PDF) [file pone.0183181.s001.pdf]

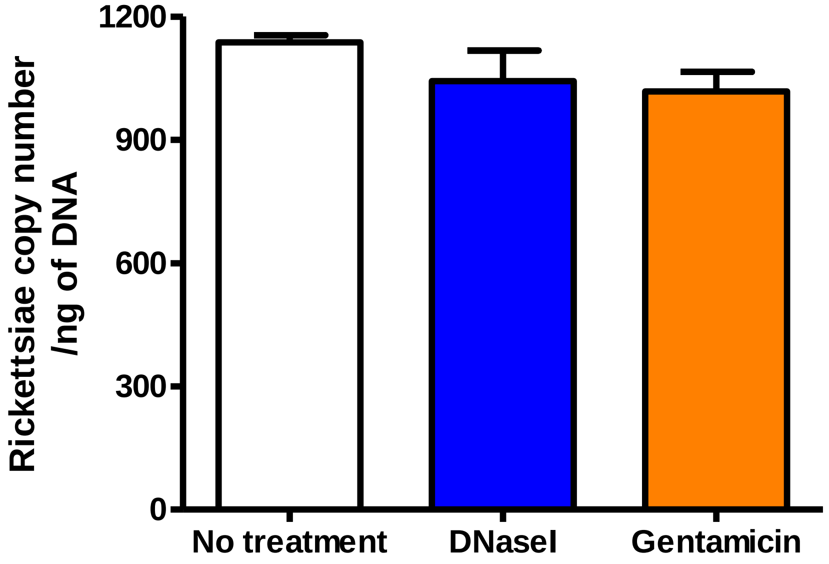

S1 Fig: Effect of DNaseI and Gentamicin on rickettsial internalization: *R. rickettsii*- infected HMECs were incubated with DNase1 (1U/ml) or gentamicin (100 µg/ml) for 30 minutes to remove any extracellular bacteria prior to the collection of infected cells for isolation of DNA and rickettsiae copy number was measured. The data represent mean ± SEM of three separate experiments.
