# Supplementary material for: Fibroblast growth factor receptor-1 mediates internalization of pathogenic spotted fever rickettsiae into host endothelium
Source: PLoS One. 2017 Aug 14;12(8):e0183181. doi: 10.1371/journal.pone.0183181 (PMC5555671; doi:10.1371/journal.pone.0183181)
Supplement: S2 Fig — (PDF) [file pone.0183181.s002.pdf]

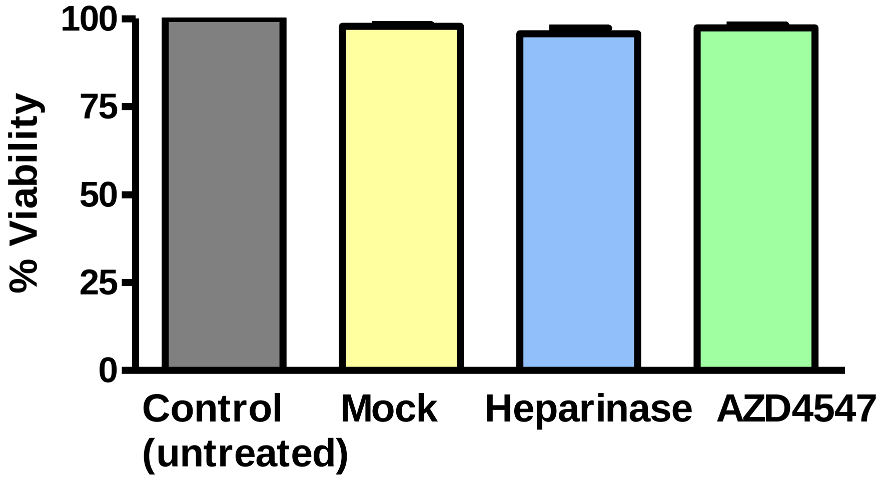

S2 Fig: Effect of Heparinase and AZD4547 on viability of endothelial cells. EC were treated with either mock or 1U/ml of heparinase or DMSO mock or 100nM AZD4547 and measured the LDH release into the culture medium by LDH assay kit (Thermo Fisher Scientific) and % viability was then determined using untreated endothelial cells as baseline control. Data represent mean ± SEM of three independent experiments performed in triplicate.
