# Supplementary material for: Fibroblast growth factor receptor-1 mediates internalization of pathogenic spotted fever rickettsiae into host endothelium
Source: PLoS One. 2017 Aug 14;12(8):e0183181. doi: 10.1371/journal.pone.0183181 (PMC5555671; doi:10.1371/journal.pone.0183181)
Supplement: S3 Fig — (PDF) [file pone.0183181.s003.pdf]

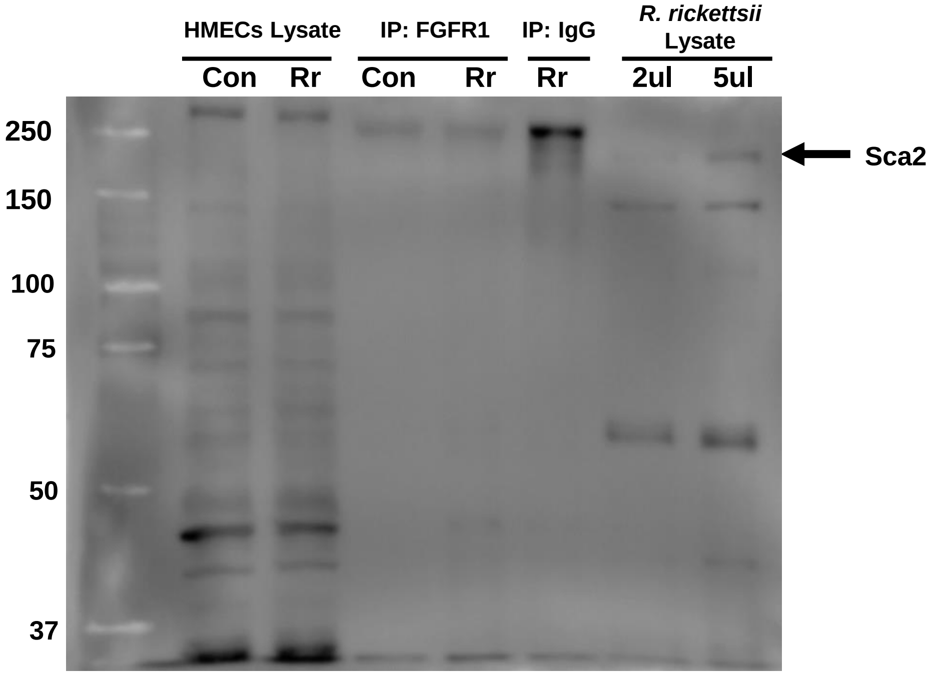

S3 Fig: Lack of Sca2 association with FGFR1: FGFR1 was immunoprecipitated (IP) from the lysates of *R. rickettsii*-infected ECs using an FGFR1-specific antibody and samples were subjected to SDS-PAGE and Western blotting using Sca2 antiserum (1:100 dilution). Mouse IgG was used as control. A representative blot is shown.
