# Supplementary figures and images for: Fibroblast growth factor receptor-1 mediates internalization of pathogenic spotted fever rickettsiae into host endothelium
Source: PLoS One. 2017 Aug 14;12(8):e0183181. doi: 10.1371/journal.pone.0183181 (PMC5555671; doi:10.1371/journal.pone.0183181)

Figure 2C

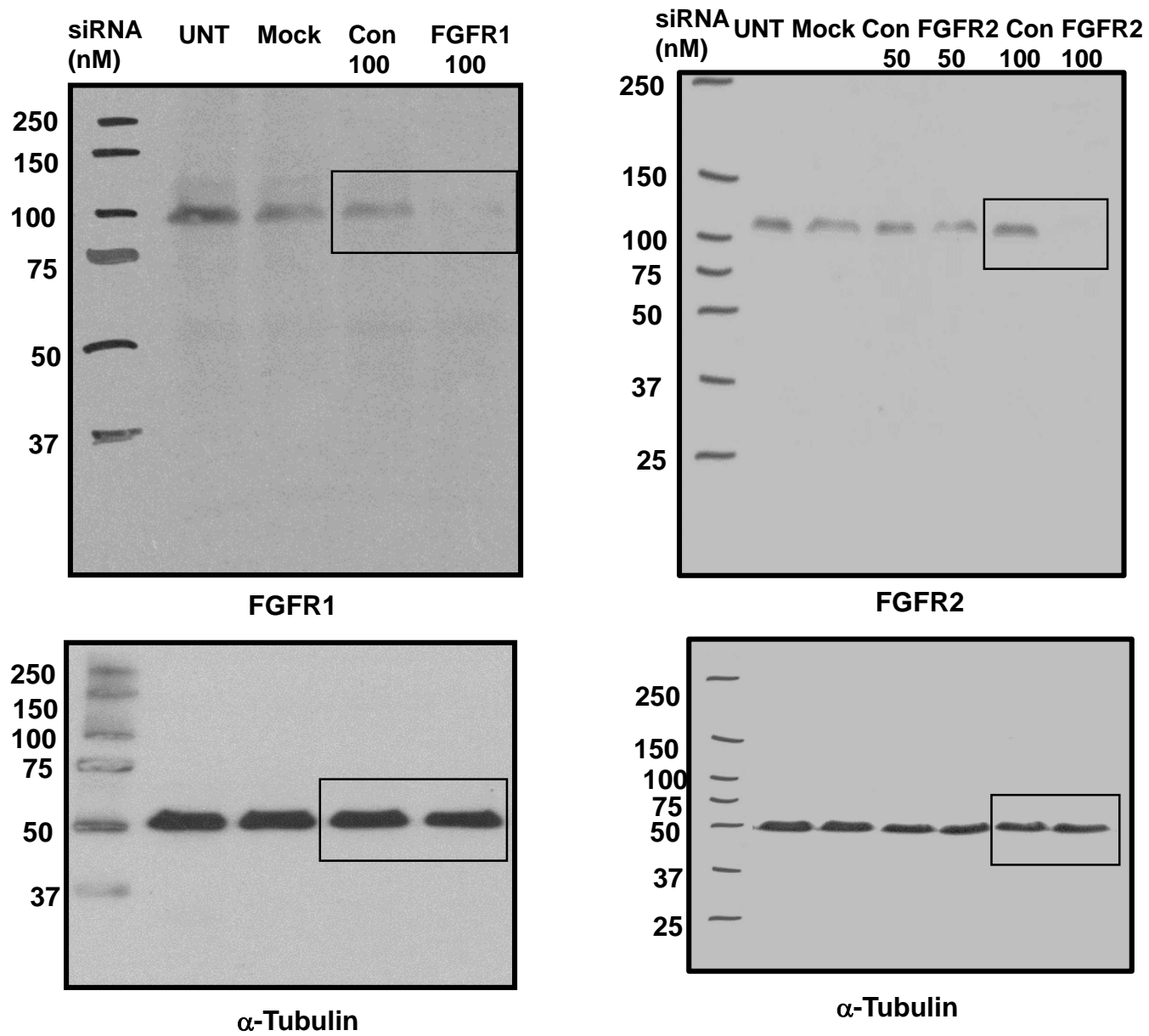

**Figure 3**

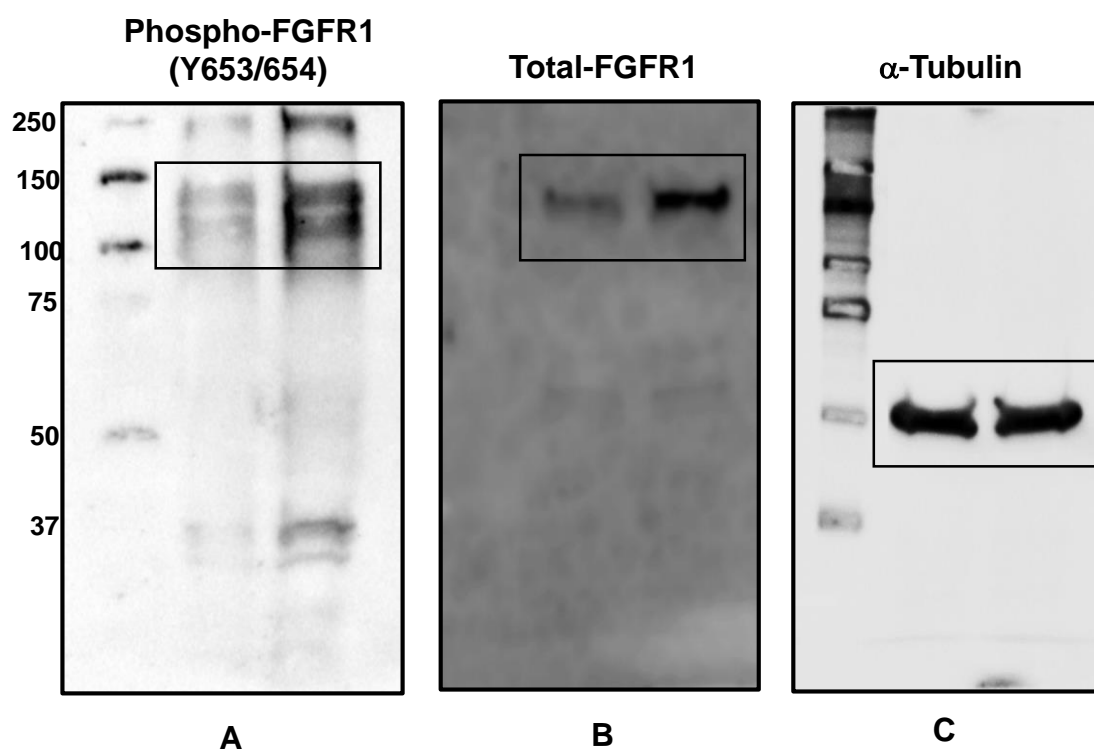

**Figure 4C**

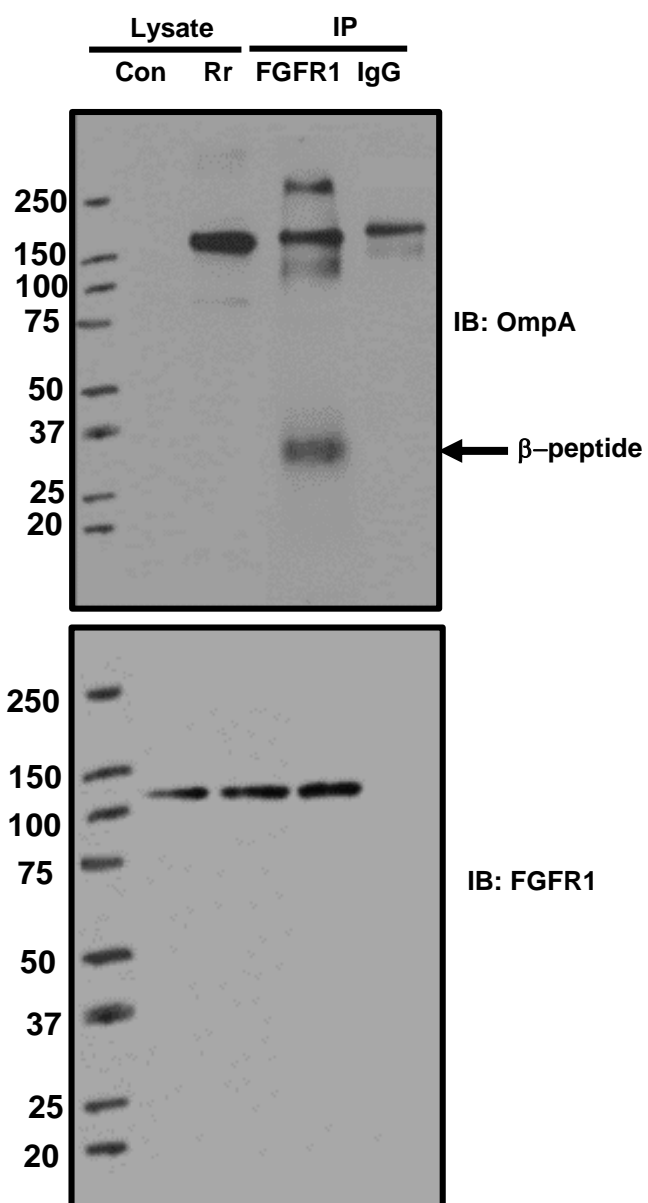

Figure 5A

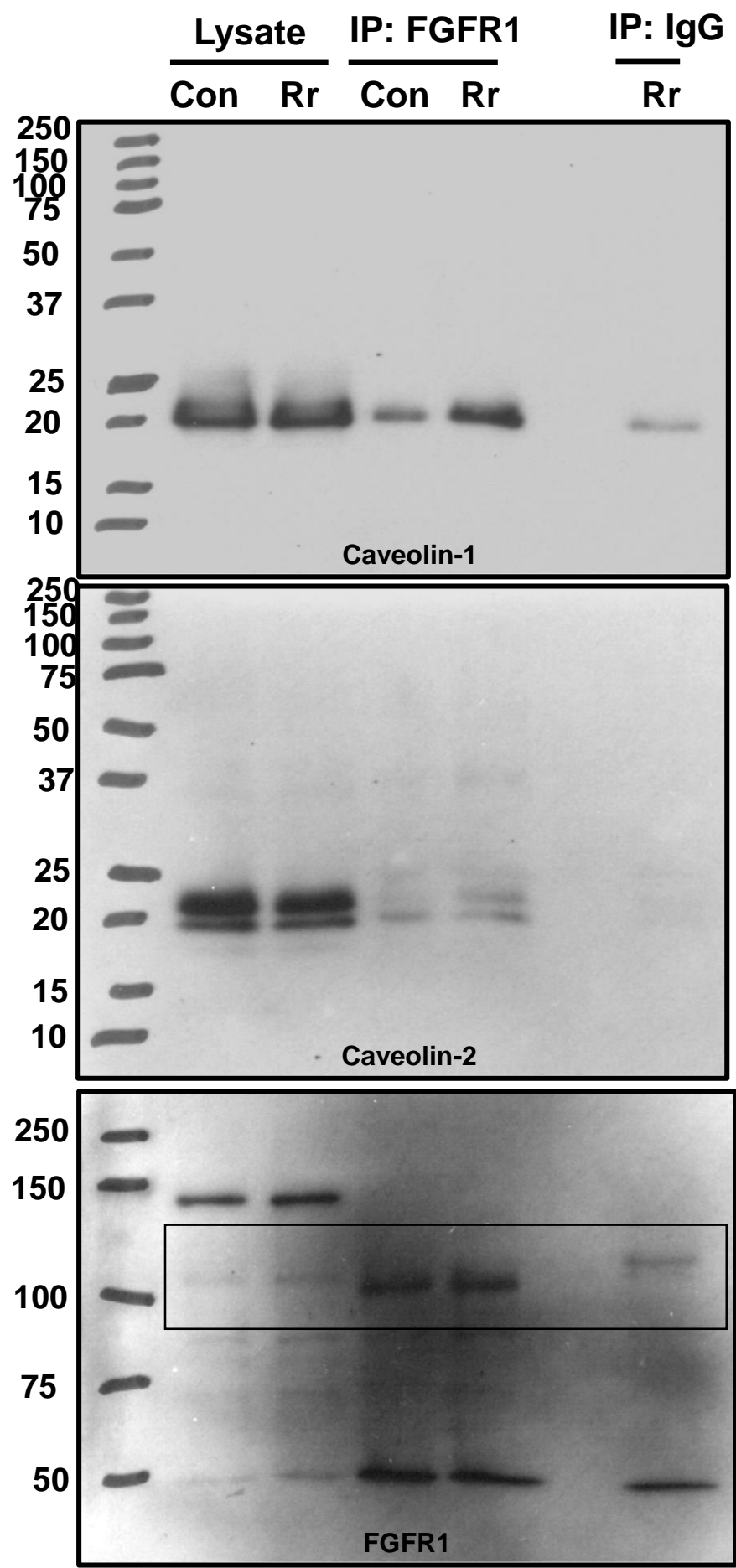

Figure 5C

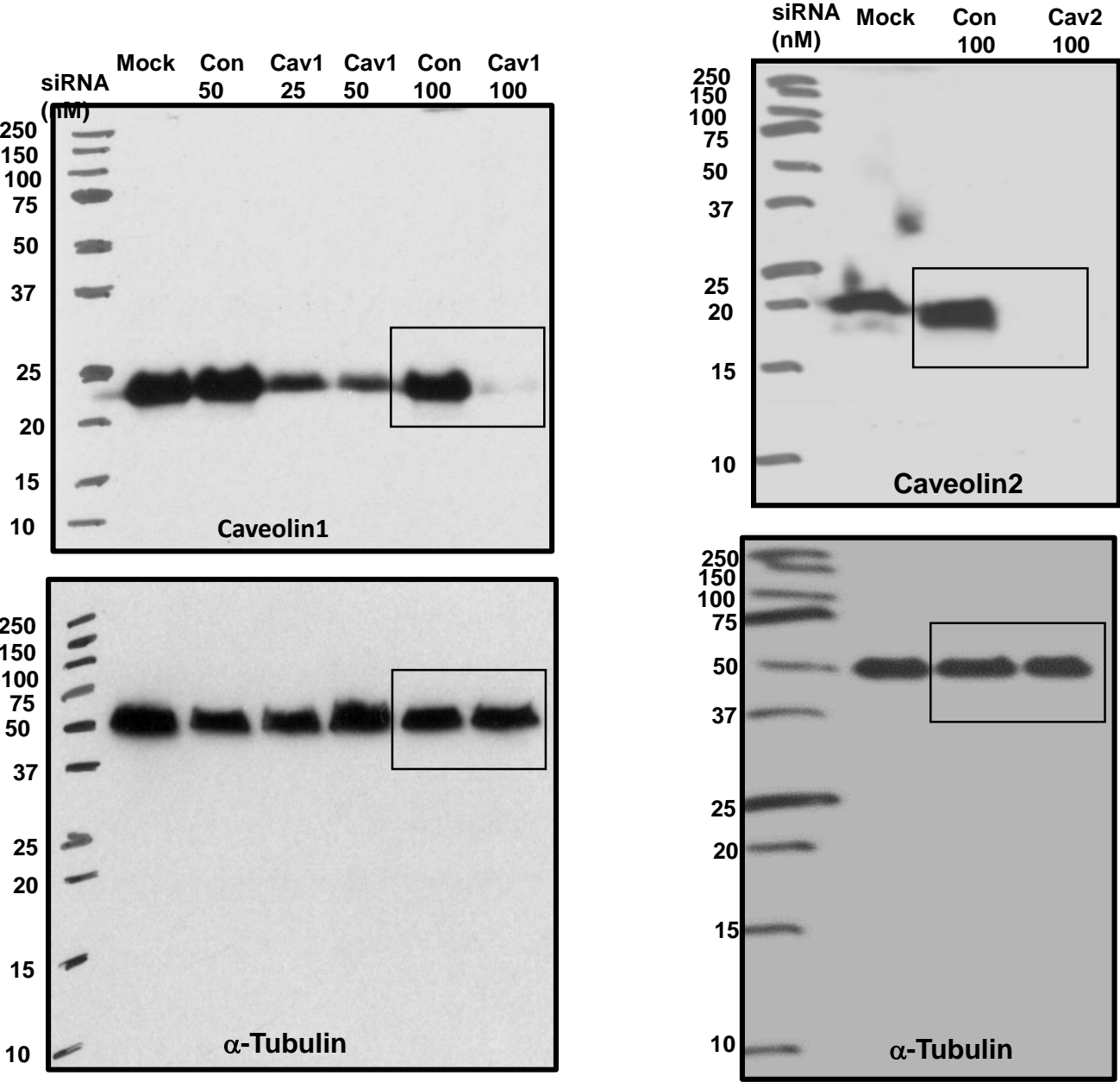

Supplement: S1 File — (PDF) [file pone.0183181.s004.pdf]
